# Supplementary material for: “What you say and how you say it” matters: An experimental evidence of the role of synchronicity, modality, and message valence during smartphone-mediated communication
Source: PLoS One. 2020 Sep 17;15(9):e0237846. doi: 10.1371/journal.pone.0237846 (PMC7497981; doi:10.1371/journal.pone.0237846)

**S1 Figure. Experimental material for both synchronous (immediate) and asynchronous (7 minutes delay) conditions: a) = with emojis, positive answers, b) text only, positive answers, c) with emojis, negative answers, d) text only, negative answers.**

a)
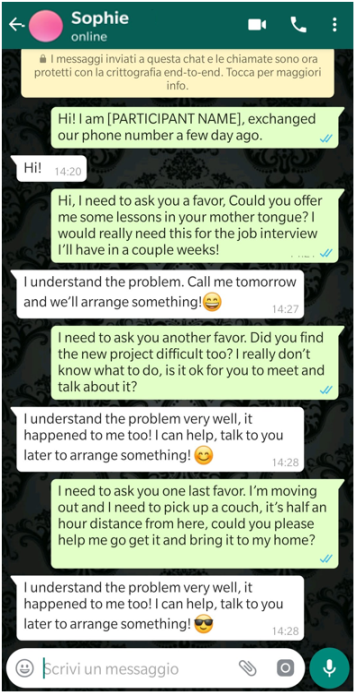
 b)
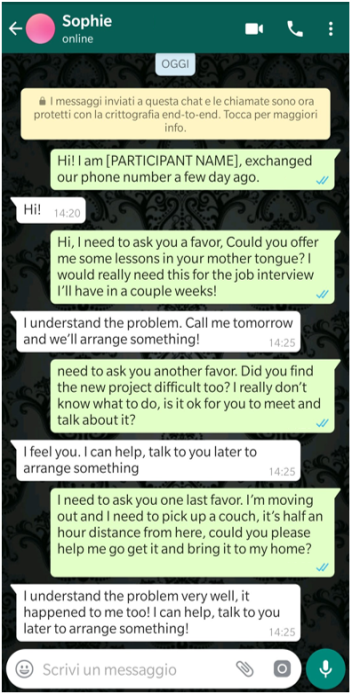


c)
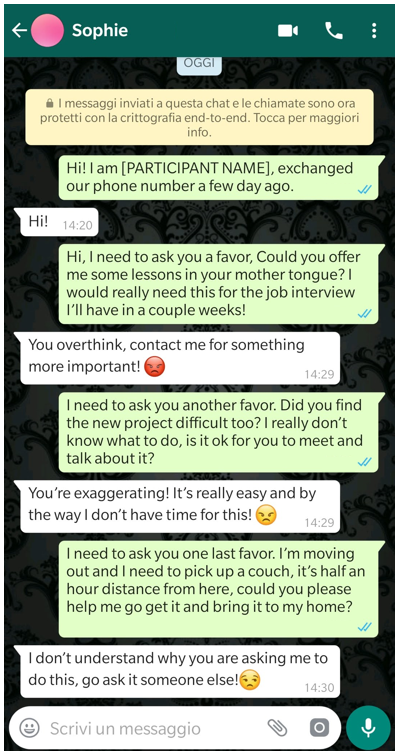
 d)
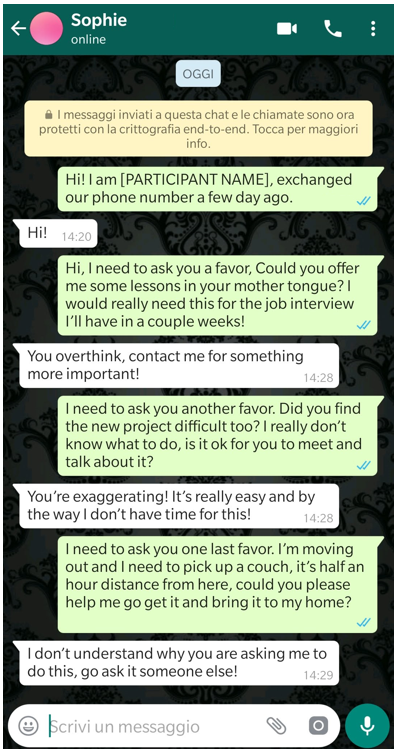

Supplement: S1 Fig — (DOCX) [file pone.0237846.s001.docx]
