# Supplementary material for: “What you say and how you say it” matters: An experimental evidence of the role of synchronicity, modality, and message valence during smartphone-mediated communication
Source: PLoS One. 2020 Sep 17;15(9):e0237846. doi: 10.1371/journal.pone.0237846 (PMC7497981; doi:10.1371/journal.pone.0237846)
Supplement: S2 Fig — Order of the favors (“language lessons”, “work/study project”, and “moving couch”) randomly chosen by the participants. (DOCX) [file pone.0237846.s002.docx]

**S2 Figure. Step-by-step procedure implemented in the study. Order of the favors (“language lessons”, “work/study project”, and “moving couch”) randomly chosen by the participants.**

Introduction

Informed consent

Introduction of scenario “new in the city” and characters (Sophie/Mark)

Favor 2

Distractor task 2

Distractor task 3

Favor 3

**Post-Questionnaire**

Social Presence

Interpersonal Trust

Social Support

Personality Attribution

Debriefing

**Pre-Questionnaire**

Socio-demographics

Favor 1

Distractor task 1
