# Supplementary material for: “What you say and how you say it” matters: An experimental evidence of the role of synchronicity, modality, and message valence during smartphone-mediated communication
Source: PLoS One. 2020 Sep 17;15(9):e0237846. doi: 10.1371/journal.pone.0237846 (PMC7497981; doi:10.1371/journal.pone.0237846)
Supplement: Attachment — Submitted filename: response to Reviewers - Plos one.docx [file pone.0237846.s005.docx]

Dear Editorial Team,

Dear Reviewers,

Please find below our answers to your suggestions/revisions.

All my best

Serena Petrocchi

PONE-D-20-10424
"What you say and how you say it” matters:  An experimental evidence of the role of synchronicity, modality, and message valence during smartphone-mediated communication
PLOS ONE

Dear Dr. Petrocchi,

Thank you for submitting your manuscript to PLOS ONE. After careful consideration, we feel that it has merit but does not fully meet PLOS ONE’s publication criteria as it currently stands. Therefore, we invite you to submit a revised version of the manuscript that addresses the points raised during the review process.

Please submit your revised manuscript by Aug 01 2020 11:59PM. If you will need more time than this to complete your revisions, please reply to this message or contact the journal office at [plosone@plos.org](mailto:plosone@plos.org). When you're ready to submit your revision, log on to <https://www.editorialmanager.com/pone/> and select the 'Submissions Needing Revision' folder to locate your manuscript file.

Please include the following items when submitting your revised manuscript:

- A rebuttal letter that responds to each point raised by the academic editor and reviewer(s). You should upload this letter as a separate file labeled 'Response to Reviewers'.
- A marked-up copy of your manuscript that highlights changes made to the original version. You should upload this as a separate file labeled 'Revised Manuscript with Track Changes'.
- An unmarked version of your revised paper without tracked changes. You should upload this as a separate file labeled 'Manuscript'.

If you would like to make changes to your financial disclosure, please include your updated statement in your cover letter. Guidelines for resubmitting your figure files are available below the reviewer comments at the end of this letter.

If applicable, we recommend that you deposit your laboratory protocols in [protocols.io](http://protocols.io/) to enhance the reproducibility of your results. Protocols.io assigns your protocol its own identifier (DOI) so that it can be cited independently in the future. For instructions see: <http://journals.plos.org/plosone/s/submission-guidelines#loc-laboratory-protocols>

We look forward to receiving your revised manuscript.

Kind regards,

Frantisek Sudzina
Academic Editor
PLOS ONE

Journal requirements:

When submitting your revision, we need you to address these additional requirements.

1. Please ensure that your manuscript meets PLOS ONE's style requirements, including those for file naming. The PLOS ONE style templates can be found at

<https://journals.plos.org/plosone/s/file?id=wjVg/PLOSOne_formatting_sample_main_body.pdf> and

<https://journals.plos.org/plosone/s/file?id=ba62/PLOSOne_formatting_sample_title_authors_affiliations.pdf>

Authors: we can confirm that the ms follow the requirements

2. We noted in your submission details that a portion of your manuscript may have been presented or published elsewhere.

"A preliminary version of the manuscript has been submitted to the 70th annual conference of the International Communication Association in 2020."

Please clarify whether this conference proceeding was peer-reviewed and formally published. If this work was previously peer-reviewed and published, in the cover letter please provide the reason that this work does not constitute dual publication and should be included in the current manuscript.

Authors: a preliminary version of the paper was submitted to the 70th annual conference of the International Communication Association in 2020. The conference proceedings are not formally published. They are available only for the participants to the conference. For this reason, the paper submitted to PlosOne does not constitute dual publication.

3. Please include captions for your Supporting Information files at the end of your manuscript, and update any in-text citations to match accordingly. Please see our Supporting Information guidelines for more information: http://journals.plos.org/plosone/s/supporting-information.

Authors: we have included a caption for our supporting information file, which now is called S1_Figure according to the journal’s guidelines.

[Note: HTML markup is below. Please do not edit.]

Reviewers' comments:

Reviewer's Responses to Questions

**Comments to the Author**

1. Is the manuscript technically sound, and do the data support the conclusions?

The manuscript must describe a technically sound piece of scientific research with data that supports the conclusions. Experiments must have been conducted rigorously, with appropriate controls, replication, and sample sizes. The conclusions must be drawn appropriately based on the data presented.

Reviewer #1: Yes

Reviewer #2: Yes

2. Has the statistical analysis been performed appropriately and rigorously?

Reviewer #1: Yes

Reviewer #2: Yes

3. Have the authors made all data underlying the findings in their manuscript fully available?

The [PLOS Data policy](http://www.plosone.org/static/policies.action#sharing) requires authors to make all data underlying the findings described in their manuscript fully available without restriction, with rare exception (please refer to the Data Availability Statement in the manuscript PDF file). The data should be provided as part of the manuscript or its supporting information, or deposited to a public repository. For example, in addition to summary statistics, the data points behind means, medians and variance measures should be available. If there are restrictions on publicly sharing data—e.g. participant privacy or use of data from a third party—those must be specified.

Reviewer #1: Yes

Reviewer #2: Yes

4. Is the manuscript presented in an intelligible fashion and written in standard English?

PLOS ONE does not copyedit accepted manuscripts, so the language in submitted articles must be clear, correct, and unambiguous. Any typographical or grammatical errors should be corrected at revision, so please note any specific errors here.

Reviewer #1: Yes

Reviewer #2: Yes

5. Review Comments to the Author

Please use the space provided to explain your answers to the questions above. You may also include additional comments for the author, including concerns about dual publication, research ethics, or publication ethics. (Please upload your review as an attachment if it exceeds 20,000 characters)

Reviewer #1: Manuscript title: "What you say and how you say it” matters: An experimental evidence of the role of synchronicity, modality, and message valence during smartphone-mediated communication

MINOR revisions
Summary:
I would like to congratulate the authors because the manuscript is really interesting and well written. It reports a well-structured mediation analysis concerning Smartphone-Mediated Communication across a sample of young individuals. This work should have a relevant impact on literature. However, in my humble opinion, some points need further attention to improve the value of the article.

Major comments:

Experimental Design:
Line 192: The Authors declare a 2X2X2 experimental design. However, it is not clear which are the conditions of the factorial design are – and how they were manipulated.

Authors: thank you for your comment. The three experimental conditions are the valence of the message (empathic accurate vs. empathic inaccurate message), the modality (with or without emojis), and the synchronicity of the message (immediately after the participant sent the request vs. 7 minutes later). To increase statistical power, we decided to collapse the conditions to be able to perform a SEM analysis and test for the hypothesized mediation of social presence. That said, we understand that the sentence can be misleading and we deleted it from the revised version of the paper.

Line 197: In addition, the Authors state: «the participants communicated with a peer»: Sophie (for females) or Mark (for males). However, the authors should explain why they did not choose for a peer sex balance which the participants communicated with.

Authors: we decided to match the gender of the peer to the gender of the participant for two main reasons. First, there is evidence showing that females and males have different communication styles during mediated communication (e.g., Savicki, Kelley, 2004) and different aims during their interactions via smartphone (e.g., Park, Lee, 2014). For this reasons, we decided to match peer’s gender and participant’s gender. If we would not have matched the gender, we should have had to control for the influence of interacting with a same-gender peer or a different-gender peer. This would have required a larger sample that, given the available resources, we could not reach. We implemented this consideration into the manuscript. Second, we wanted to avoid any romantic connotations. You can already find this consideration in the manuscript (see line 197).

Line 204: the Authors introduce a “Pilot study”. Unfortunately, it’s not completely clear if both the procedure (line 235), measures (262) refers to the “Main study” or the “pilot study” – probably because there are no results reported from this “pilot study” (but are available on request). Thus, probably it could be helpful to provide some results of this pilot study.

Authors: the part of the text from line 211 to line 245 pertains to the pilot study with the information that detailed results of the pilot study are available upon request (we added “detailed” in the manuscript, line 232). The part from line 247 to line 397 pertains to the experimental study. We clarified this point now including a specification in the titles of the Procedure and Measures sections and a specification in line 345 regarding study participants.

Data Analysis:

Table 1: some correlation values suggest the presence of multicollinearity (e.g.: variable#7 and variable#2). Is it correct? Moreover, it’s not completely clear why correlations between variable#5, #6, and #7 are not applicable (n/a).

Authors: many thanks for this important observation. In fact, the presence of multicollinearity depended on the shared variance due to the three experimental manipulation variables that added a common variance to the correlations between the variables. We calculated a new table of partial correlations controlling for the three conditions (i.e., valence, modality, and synchronicity). As you can see now, the correlations are lower than before suggesting an association between construct but not an overlap or signs of multicollinearity.

The n/a was added because the correlations between the three experimental conditions are meaningless, so in this sense “not applicable”. We acknowledge that including these three variables in the table could mislead a reader. Thus, we decided to delete them from the table.

Discussion:
The discussion section should be implemented by a deepened analysis of the results across the theoretical framework.

Moreover, the discussion section should be adjusted considering previous comments.

Authors: we checked and adjusted our discussion based on the revisions of the previous sections of our manuscript. Based on the suggestions by Reviewer 2, we have also expanded on the practical and scientific implications of our study in the conclusions section.

Minor comments:
Line 6: it is not immediately understandable what SNC refers to. The first time a concept is introduced it should be avoided to use only its acronym.

Authors: we have specified the acronym in the abstract where first time it is mentioned.

Line 67-73: please, add at least one reference.

Authors: we did, thank you

Line 83-90: please, add at least one reference.

Authors: we did, thank you

Line 135-139: please, add at least one reference.

Authors: we did, thank you

Table 2: table 2 is not easy to interpret and could be better structured.

Authors: thank you. We slightly changed the layout of the table and hope that it is now easier to read interpret.

Figures 1 and 2: both figures are too small and not easy to read – and they don't do justice to such beautiful research work. Could it be possible to propose a more suitable graphic version?

Authors: thank you for your comment, we enlarged the figures.

Reviewer #2: The present experimental study aimed at investigating the impact of three aspects of SMC, i.e. synchronicity, modality, and valence, on interpersonal trust, social support, and personality attribution, through the mediation of perceived and subjective social presence of the communication partner.
The approach used for experimental manipulation is innovative and of scientific interest.
However, I suggest strengthening some aspects, above all of a theoretical nature, in order to make the study ready for publication


1. In the abstract I would spend a few more words to explain the constructs and variables (not in detail), while I would reduce the first sentence which is not specifically related to this study “Although past research has investigated the differential effects of online and offline communication on psychosocial functioning, smartphone-mediated-communication and instant messaging are fast-changing environments in which technological advancements spread quickly and widely in the population.”

Authors: thank you for your comment. We have deleted the first sentence and explained in more details what are the three experimental conditions, the mediators, and the expected outcomes.

2. Also in the introduction I would cut the part about the historical evolution of technologies while I would explain the importance of this study. Why is it important to study the relationship between the variables investigated? From the premises to the goal of the study there is a jump (“This experimental study aims to contribute to scientific evidence on the role of specific aspects of SMC (i.e. synchronicity, modality, and message valence) in the perception of the communication partner (i.e. social presence) and the quality of the interpersonal relationship in the context of SMC.

Before introducing the study, the scientific literature on SMC and related concepts will be reviewed to derive a theoretical model guiding the experimental study and the interpretation of its results.”). I suggest to better focus the variables that will be manipulated and tested before stating the goal of the study

Authors: we cut the historical evolution of technologies leaving only the part that really matters with our study and is important as introduction to the study of SMC. We better explained what is the contribution of our study and introduced the variables under analysis.

3. Please better explain how did you manipulate the empathic accuracy. At page 13 you stated “Empathic accuracy is defined as the degree to which a person infers what other people think and feel [38,39], enhancing the experience of positive or negative feelings and emotions.” I do not understand why positive valence is considered an expression of empathic accuracy (vs. negative valence, i.e. empathic inaccuracy)

Authors: please see page 13. We have better defined what empathic accuracy means considering the literature and the context of our research. *Empathic accuracy is defined as the degree to which a person infers what other people think and feel [38,39], enhancing the experience of positive or negative feelings and emotions. In this vein, an empathic accurate answer to a request is a message in which the sender takes into consideration the receiver’s mental state (i.e., what he/she thinks and how he/she feels) and replies accordingly, thus creating a positive experience*. Based on this definition, an answer that takes into account the thoughts and feelings of the receiver is an empathic accurate message and has a positive valence. Contrarily, an answer that does not take into account the thoughts and feelings of the receiver is an empathic inaccurate message and has a negative valence.

4. I suggest to include a figure representing the experimental design to facilitate readers in understanding the procedure and study design

Authors: please find the flowchart we have created.

5. the “pilot” study seems more a “preliminary” study. This preliminary study should be introduced before in the text. Did you involve the same participants?

Authors: the pilot study involved different participants than those that have been involved in the main experimental study. The main aim of the pilot study was to develop and evaluate the experimental material, i.e. the scenarios and the responses in a simulated context of smartphone-mediated communication to be able to test our research hypotheses. More precisely, in the pilot study, we wanted to understand which scenarios were the most plausible, frequent, and realistic for our social context given the fact that we adapted an experimental procedure developed by Feng et al (2004). Moreover, we wanted to test whether the developed responses were perceived as empathic (or non-empathic). As specified in the manuscript, we approached a separate sample for this pilot study. We specified this aspect now in the manuscript in the paragraph titles “Experimental Study Participants”. Since the pilot has different participants than the main study but has research questions related to the main study, we refer to it as a pilot study instead of preliminary study.

6. Discussion. The relevance of the study and implications should be enhanced

 Authors: thank you for your comment. We revised the discussion to highlight better the implications of our research and its contribution to further develop research in the field of smartphone-mediated communication.

6. PLOS authors have the option to publish the peer review history of their article ([what does this mean?](https://journals.plos.org/plosone/s/editorial-and-peer-review-process#loc-peer-review-history)). If published, this will include your full peer review and any attached files.


If you choose “no”, your identity will remain anonymous but your review may still be made public.


**Do you want your identity to be public for this peer review?** For information about this choice, including consent withdrawal, please see our [Privacy Policy](https://www.plos.org/privacy-policy).

Reviewer #1: No

Reviewer #2: Yes: Daniela Villani


[NOTE: If reviewer comments were submitted as an attachment file, they will be attached to this email and accessible via the submission site. Please log into your account, locate the manuscript record, and check for the action link "View Attachments". If this link does not appear, there are no attachment files.]

While revising your submission, please upload your figure files to the Preflight Analysis and Conversion Engine (PACE) digital diagnostic tool, <https://pacev2.apexcovantage.com/>. PACE helps ensure that figures meet PLOS requirements. To use PACE, you must first register as a user. Registration is free. Then, login and navigate to the UPLOAD tab, where you will find detailed instructions on how to use the tool. If you encounter any issues or have any questions when using PACE, please email PLOS at [figures@plos.org](mailto:figures@plos.org). Please note that Supporting Information files do not need this step.
